# Glutaminase Increases in Rat Dorsal Root Ganglion Neurons after Unilateral Adjuvant-Induced Hind Paw Inflammation

**DOI:** 10.3390/biom6010010

**Published:** 2016-01-13

**Authors:** E. Matthew Hoffman, Zijia Zhang, Ruben Schechter, Kenneth E. Miller

**Affiliations:** Anatomy and Cell Biology, Oklahoma State University Center for Health Sciences, 1111 West 17th Street, Tulsa, OK 74107, USA; e.matthew.hoffman@gmail.com (E.M.H.), zijia.zhang@okstate.edu (Z.Z.)

**Keywords:** glutaminase, glutamate, adjuvant-induced arthritis, complete Freund’s adjuvant, dorsal root ganglion

## Abstract

Glutamate is a neurotransmitter used at both the peripheral and central terminals of nociceptive primary sensory neurons, yet little is known concerning regulation of glutamate metabolism during peripheral inflammation. Glutaminase (GLS) is an enzyme of the glutamate-glutamine cycle that converts glutamine into glutamate for neurotransmission and is implicated in producing elevated levels of glutamate in central and peripheral terminals. A potential mechanism for increased levels of glutamate is an elevation in GLS expression. We assessed GLS expression after unilateral hind paw inflammation by measuring GLS immunoreactivity (ir) with quantitative image analysis of L4 dorsal root ganglion (DRG) neurons after one, two, four, and eight days of adjuvant-induced arthritis (AIA) compared to saline injected controls. No significant elevation in GLS-ir occurred in the DRG ipsilateral to the inflamed hind paw after one or two days of AIA. After four days AIA, GLS-ir was elevated significantly in all sizes of DRG neurons. After eight days AIA, GLS-ir remained elevated in small (<400 µm^2^), presumably nociceptive neurons. Western blot analysis of the L4 DRG at day four AIA confirmed the elevated GLS-ir. The present study indicates that GLS expression is increased in the chronic stage of inflammation and may be a target for chronic pain therapy.

## 1. Introduction

Central axons of pseudounipolar primary sensory neurons of the dorsal root ganglion (DRG) terminate in spinal cord dorsal horn and medullary dorsal column nuclei for pain and touch pathways, respectively [1]. Peripheral axons of these neurons terminate in and bring sensory information from target tissues, such as viscera, muscle and skin. The central axon terminals release the neurotransmitter glutamate at both spinal [2,3,4,5] and medullary levels [2]. Although the peripheral axon terminals are afferent receptors, some of them also have efferent capabilities [6,7], e.g., glutamate release in response to noxious stimulation [8,9,10]. Inflammation of peripheral target tissues increases the amount of glutamate released from both the peripheral [9,10] and central axon terminals of DRG neurons [3,4]. Similar phenomena occur with the neuropeptides substance P (SP) and calcitonin gene-related peptide (CGRP) when inflammation induces gene regulation at the transcriptional level, providing the elevated amounts of SP and CGRP for release from peripheral and central terminals [11,12]. Increasing the quantity of glutamate available for release depends on regulating the amount of the enzyme for synthesizing glutamate, *i.e.*, glutaminase (GLS; EC 3.5.1.2). Despite glutamate being released from the same neurons that release neuropeptides [13] and the co-localization of GLS within peptidergic neurons [14], very little is known about glutamate metabolism in DRG neurons during inflammation. One study indicates that GLS is elevated in small and medium diameter lumbar 4 (L4) DRG neurons after seven ys of unilateral adjuvant-induced arthritis (AIA) [15].

Glutamate metabolism in the central nervous system (CNS) has been well studied [16], since glutamate is the primary excitatory neurotransmitter in the CNS. Neurotransmitter glutamate is taken up and converted to glutamine by astrocytes via the enzyme glutamine synthetase, which release glutamine back to the neurons. Neurons take up glutamine and convert it to glutamate with GLS. Many of the proteins necessary for the glutamate-glutamine cycle are present in DRG neurons and their glia [7,17,18,19,20,21]. Acute increase in the production of glutamate available for release in DRG neurons could involve an increase in flux through the glutamate-glutamine cycle near the sites of glutamate release. Long-term responses may require regulating the expression of glutamate-glutamine cycle proteins at the cell body in the DRG and then transporting them to the peripheral and central terminals [7,15]. To address this issue, we hypothesize that hind paw inflammation increases GLS production in rat DRG neurons. In the present study, we examined GLS-immunoreactivity (ir) with quantitative image analysis of neurons in the rat lumbar 4 (L4) DRG neurons after one, two, four, and eight days of hind paw inflammation, *i.e.*, AIA, and Western blot after four days of AIA.

## 2. Materials and Methods

### 2.1. Animals

A combination of male and female Sprague-Dawley rats (*n* = 32; 170–280 g) bred on site were used for this study. They were housed on a 12 h light: 12 h dark cycle and given free access to food and water. Procedures in this study were conducted according to guidelines from the International Association for the Study of Pain [22] and the National Institutes of Health [23], and were approved by the Oklahoma State University—Center for Health Sciences Institutional Animal Care and Use Committee (Protocol # 2010-02). All appropriate efforts were made to minimize the number of rats used in this study.

To induce a unilateral inflammation of the hind paw, rats (*n* = 20) were anesthetized with isoflurane and 150 µL of a 1:1 emulsion containing complete Freund’s adjuvant (CFA; Sigma; St. Louis, MO, USA) and 10 mM phosphate buffered 0.9% saline (PBS) was injected into the plantar surface of the right hind paw using a 26 gauge needle. Control rats (*n* = 12) were given an injection of 150 µL PBS in the right hind paw with the same anesthesia procedure and injection technique. Inflammation was allowed to persist for one, two, four, or eight days; at each time point, 5 CFA-injected and 3 PBS-injected animals were used for immunohistochemistry experiments.

### 2.2. Evoked Pain Behavioral Responses

Behavioral studies were performed to verify the presence of hyperalgesia after induction of inflammation. Rats were housed in a behavioral testing room within the animal facility to familiarize them to the testing environment and to minimize the experience of transfer to and from testing chambers and housing cages. Three days of testing were performed prior to the injection day to obtain a baseline reading for each animal; these days were noted as days -3, -2, and -1. On injection day (day 0), the behavioral test was done prior to the injection, thus serving as a fourth and final assessment of baseline sensitivity. Behavioral testing continued daily at the same approximate time each day throughout the remainder of each individual experiment, with the longest being until day 8 (12 total days of testing).

Thermal latencies measured in seconds were obtained using a Plantar Test apparatus (Ugo Basile, Comerio, Italy) set at an intensity of 55 mW/cm^2^. Mechanical thresholds measured in grams were obtained using a Dynamic Plantar Aesthesiometer (Ugo Basile) set to apply a maximum of 50 g at a ramp rate of 5 g/s. Each testing period consisted of placing the rats into the testing chambers where acclimation was indicated by cessation of all exploratory and grooming behaviors. Two thermal latencies and two mechanical thresholds were measured from each hind paw of each rat, with measurements spaced at least ten minutes apart.

### 2.3. Hind Paw Edema

Hind paw edema was measured with a dial caliper (Mitutoyo; Aurora, IL, USA) by measuring metatarsal thickness to the nearest 0.05 mm of both hind paws on the day that the rat was perfused. All hind paw edema measurements were taken when animals were 47–48 days old so that age would not confound the results.

### 2.4. Immunohistochemistry (IHC)

After one, two, four, or eight days of inflammation, 3 PBS-injected and 5 CFA-injected rats were anesthetized with intraperitoneal (i.p.) injections of 1.5 mL 2.5% (*w*/*v*) Avertin followed by 0.5 mL xylazine. Rats were perfused with 75 mL of calcium-free Tyrode’s solution, pH 7.3 followed by 325 mL of 0.96% (*w*/*v*) picric acid and 0.2% (*w*/*v*) formaldehyde in 0.1 M sodium phosphate buffer, pH 7.3. We chose this fixative because low aldehyde concentration results in optimal immunolabeling of GLS [18,24]. The right (ipsilateral) and left (contralateral) L4 DRG were carefully dissected and placed in the same fixative for 24 h at 4 °C before being transferred to 20% (*w*/*v*) sucrose in 0.1 M sodium phosphate buffer, pH 7.3 for 48 h at 4 °C. The eight DRG from a single time set of animals were embedded in a single frozen block and cut in 10 µm sections on a Microm HM 550 OMVP cryostat (Richard Allan Scientific; Kalamazoo, MI, USA). Every fourth section was thaw mounted on gelatin-coated SuperFrost slides (Fischer Scientific; Pittsburg, PA, USA) with two sections per slide. Five slides of DRG sections from each time point were dried at 37 °C for two hours. After three 10 min rinses in PBS, DRG sections were blocked for one hour at room temperature in 10% (*v*/*v*) normal goat serum, 10% (*v*/*v*) fetal bovine serum, 10% (*v*/*v*) normal horse serum, 2% (*w*/*v*) polyvinylpyrollidone, 2% (*w*/*v*) bovine serum albumin, and 0.3% (*v*/*v*) Triton X-100 in PBS. A polyclonal rabbit antiserum against glutaminase was a generous gift from Dr. Norman Curthoys (Colorado State University, Ft. Collins, CO, USA) and previous absorption controls on DRG tissue sections have been performed [18]. The primary antiserum was diluted 1:10,000 in PBS containing 0.3% (*w*/*v*) Triton X-100 (PBS-T) and DRG sections were incubated for four days at 4 °C [18]. After primary antiserum incubation, DRG sections were rinsed three times for 10 min in PBS and incubated for one hour at room temperature in biotinylated goat anti-rabbit (Vector Laboratories; Burlingame, CA, USA) diluted in PBS-T to 1.5 µg/mL. DRG sections were rinsed two times in PBS for 10 min and one time in 0.1 M sodium carbonate buffered 0.9% (*w*/*v*) saline (SCBS) for 10 min before one hour of incubation at room temperature in fluorescein isothiocyanate conjugated avidin (Vector Laboratories) diluted to 1 µg/mL in SCBS. After three 10 min rinses in PBS, cover slips were affixed with ProLong Gold (Invitrogen; Carlsbad, CA, USA) to retard fading of immunofluorescence.

### 2.5. Quantitative Image Analysis

Images of L4 DRG tissue sections were acquired on a BX51 epifluorescence microscope (Olympus; Center Valley, PA, USA) using a SPOT RT740 camera (Diagnostic Instruments; Sterling Heights, MI, USA). An exposure and gain combination was determined empirically for each of the four slide sets in which the dimmest regions of tissue could be discerned visually for tracing, but the brightest regions were not oversaturated [15,18,25,26]. Three fields of view were captured randomly from each section of each DRG. All nucleated cells were analyzed in ImageJ (National Institutes of Health; Bethesda, MD, USA) by using the freehand selection tool to identify the cells as regions of interest (ROIs). Once all ROIs for a given image were selected and added to the ROI manager, the area (in µm^2^) and cytoplasmic mean gray values were measured for each cell and exported for subsequent statistical analysis. Each pixel of an 8-bit grayscale image had a value from 0–255. Each mean gray intensity (MGI) value (C) was converted into a relative MGI value that ranged from 1–100 [27], because images from each of the four slide sets, *i.e.*, different days of inflammation, were taken at slightly different gain and exposure settings. To accomplish this conversion, the MGI value of the most weakly labeled neuron in each data set (A) and the MGI value of the most intensely labeled neuron in each data set (B) were determined and each neuron was given a relative MGI value = (100 × (C − A)/(B − A)). MGI values were used to quantitate the immunoreactivity (ir) and therefore estimate protein expression [26]. Frequency distributions of MGI’s were generated for each time point. At each of the four time points assessed, the mean MGI’s were calculated for small (<400 µm^2^), medium (400–600 µm^2^), and large (>800 µm^2^) neurons of each L4 DRG as described in previous reports [18,25,27,28].

### 2.6. Western blot

Tissues were obtained from six female Sprague-Dawley rats weighing 200–250 g; three controls were injected with PBS and three injected with CFA as described in Section 2.1. Rats were asphyxiated with CO_2_ after four days of AIA, and the right L4 DRG’s were rapidly extracted. DRG’s were homogenized with lysis buffer (pH 7.4, 10 mM Tris-HCl, 2 mM EDTA, 0.1% Triton X-100, 1% Cocktail I, 2% Cocktail II and protease inhibitor (all from Sigma-Aldrich; St. Louis, MO, USA) as previously described [15,29]. Samples were centrifuged for 20 min at 70,000 RPM at 4 °C. Supernatants were collected and total protein concentration was evaluated using bicinchoninic acid (BCA kit, Thermo Scientific; Rockford, IL, USA). Samples for GLS purification were normalized to 80 µg/mL of total protein. Samples were exposed to M-280 magnetic beads (Invitrogen; Grand Island, NY, USA) conjugated with rabbit anti-GLS antibody (gift from Dr. N.P. Curthoys) against a synthetic GLS peptide [30] in lysis buffer at 4 °C overnight as previously reported [15,31]. The GLS-bound beads were extracted with a magnet, resuspended in 10 mM Tris Base, 1 mM EDTA, 2.5% SDS, 5% β-mercaptoethanol and 0.01% bromophenol blue and boiled at 100 °C for 3 min. After a second exposure to the magnet, the supernatant containing GLS was removed and the beads were discarded. Samples and a protein molecular weight marker (Novagen; Rockland, MA, USA) were separated using the PhastSystem (GE Healthcare Life Sciences; Pittsburgh, PA, USA) employing a 12.5% homogenous SDS gel (GE Healthcare Life Sciences). The PhastSystem, using parameters previously described [15,29], was used to transfer samples to a nitrocellulose membrane (Whatman, GE Healthcare; Piscataway, NJ, USA). Membranes were dried for 10 min and blocked for 30 min with 1% bovine serum albumin (BSA) in Tris-buffered saline Tween (TBST, 20 mM Tris-HCl, 150 mM NaCl, 0.05% Tween 20, pH 7.5) at room temperature. After rinsing in TBS, the membranes were incubated overnight at 4 °C with GLS antibody at 1:1000 in TBST. Membranes were washed in TBST and incubated in secondary mouse anti-rabbit IgG alkaline phosphatase conjugate (Promega; Madison, WI, USA) at 1:7000 and S-AP conjugate at 1:5000 (Novagen; Rockland, MA, USA) for 30 min. Detection was performed using a Western Blue stabilized substrate for alkaline phosphatase (Promega; Madison, WI, USA). Densitometric analysis of Western blot images was performed with ImageTool (UTHSCSA; San Antonio, TX, USA) as described previously [15].

### 2.7. Statistical Analysis

Graphs and statistical calculations were performed in GraphPad Prism version 5.01 for Windows (GraphPad Software Inc.; San Diego, CA, USA). Two-way ANOVA was performed to determine if effects of inflammation or time were significant on thermal latency, mechanical threshold, and hind paw edema data. Bonferroni post-tests were performed to determine which groups differed and when. A two way ANOVA was performed for the GLS-ir data to determine if effects of inflammation were significant on the mean GLS-ir of small, medium, and large L4 DRG neurons. Bonferroni post-tests were performed to determine which DRG and which neuron populations differed significantly in GLS-ir. All graphical results represent mean plus or minus the standard deviation. Results were considered significant when p values were less than 0.05.

Western blot results were reported as mean density and standard error of the mean (SEM). A Student’s t test was performed on GLS Western blot data and *p* < 0.05 was considered statistical significant. The percent changes of control and AIA treated animals also were reported.

## 3. Results

### 3.1. Evoked Pain Behavioral Responses

**Figure 1:**
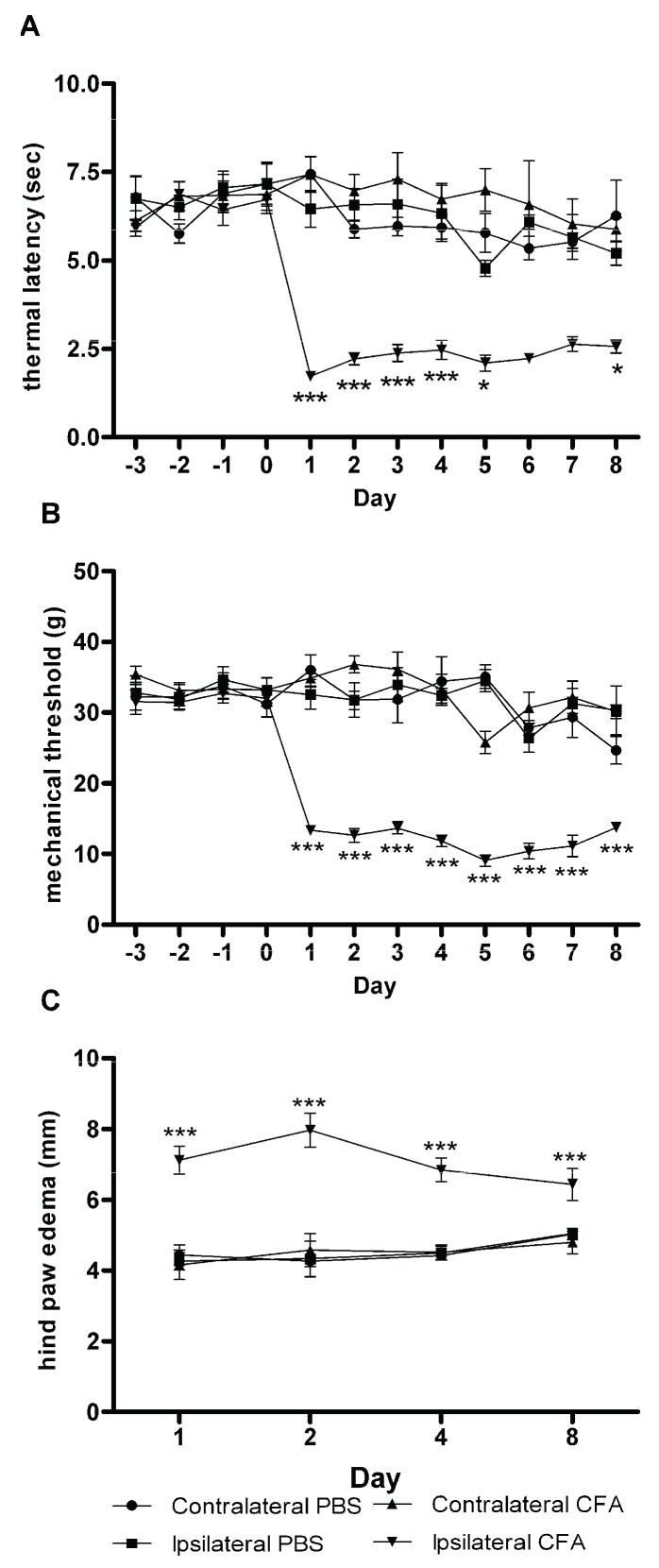
Thermal latencies, mechanical thresholds, and metatarsal thicknesses from rat hind paws after injection with phosphate buffered saline (PBS) or complete Freund’s adjuvant (CFA). Adjuvant-induced arthritis (AIA) caused significant decreases in thermal latencies (**A**) for days 1 through 4 and mechanical thresholds (**B**) for days 1 through 8. Thermal latencies appeared decreased for days 5 through 8, but significance could not be shown with sample sizes of *n* = 3 for PBS and *n* = 5 for CFA at these time points. The behavioral data from all four sets were combined; therefore, the number of rats contributing to the data set declined as the study progressed. Edema measurements (**C**) for each time point, however, consisted of 3 PBS-injected rats and 5 CFA-injected rats. At all time points, there was a significant increase in edema in the CFA-injected hind paw, with peak swelling occurring after two days of inflammation. Data are presented as mean ± SEM. * *p* < 0.05; *** *p* < 0.001.

Thermal latencies and mechanical thresholds of the hind paws ipsilateral or contralateral to the PBS injection, or contralateral to the CFA injection were never significantly different from each other. The baseline thermal latencies of the hind paws ipsilateral to the CFA injection were significantly different from the three other groups after the CFA injection at day 0 through day 4. Hind paw thermal latencies ipsilateral to the CFA injection were not different than those from hind paws ipsilateral to the PBS injection on days 5, 7 or 8 (Figure 1A). Baseline mechanical thresholds of the hind paws ipsilateral to the CFA injection were not significantly different than the other baseline measurements, but they were always significantly different from the three other groups after the injection at day 0 through day 8 (Figure 1B).

### 3.2. Hind Paw Edema

Metatarsal thicknesses of the PBS injected hindpaws were not significantly different from the hindpaws contralateral to PBS and CFA injections at any of the four time points. Metatarsal thicknesses were increased significantly (*p* < 0.001) in the hind paws ipsilateral to CFA injection compared to the other three groups of hind paw at all four time points with a peak increase at 2 days (Figure 1C). The relative increases of the CFA-injected paw *vs.* the PBS-injected paw were 67%, 84%, 52%, and 28%, for days 1, 2, 4, and 8, respectively.

### 3.3. Changes in GLS-ir During Inflammation

**Figure 2:**
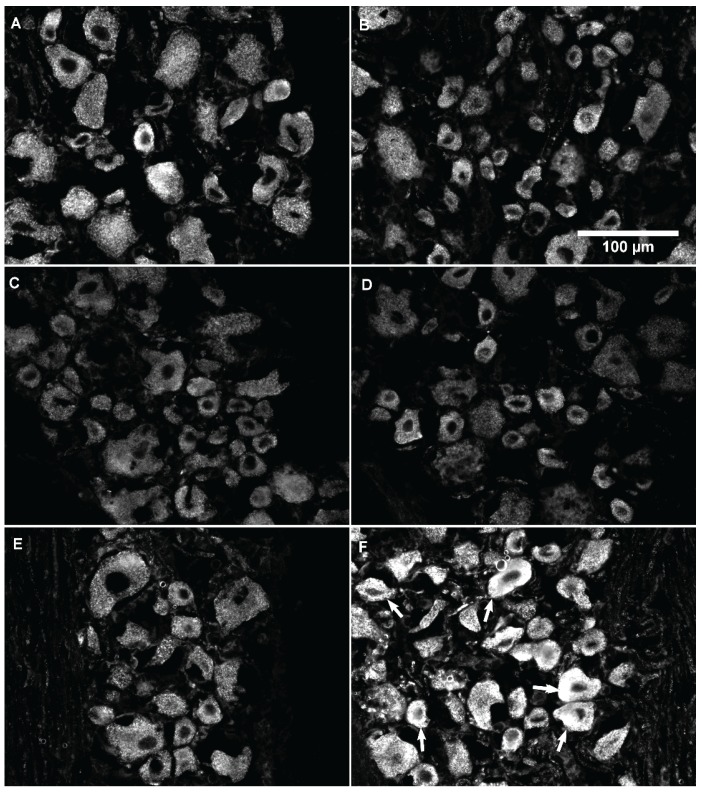

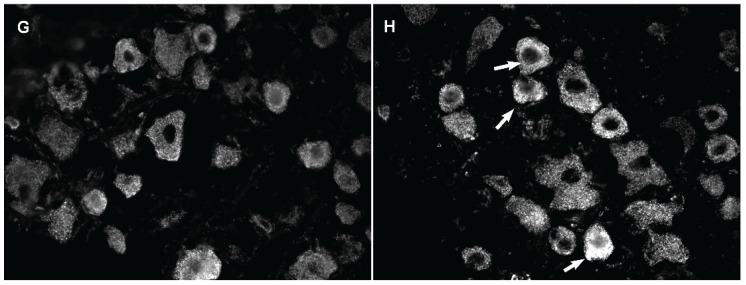
Representative images of glutaminase (GLS) immunoreactivity (ir) in lumbar 4 (L4) dorsal root ganglion (DRG) neurons ipsilateral to the PBS and CFA injected hind paws after one (**A**,**B**); two (**C**,**D**); four (**E**,**F**); and eight (**G**,**H**) days of AIA. Left column images are from PBS injected rats. Right column images are from CFA injected rats. All sizes of DRG neurons have GLS-ir. (**F**) After four days of hind paw inflammation, GLS-ir increased (arrows) in many DRG neurons, regardless of neuronal size, ipsilateral to CFA injection compared to PBS injected controls. (**H**) GLS-ir was still elevated in some small neurons (arrows) after eight days of inflammation compared to controls.

**Figure 3:**
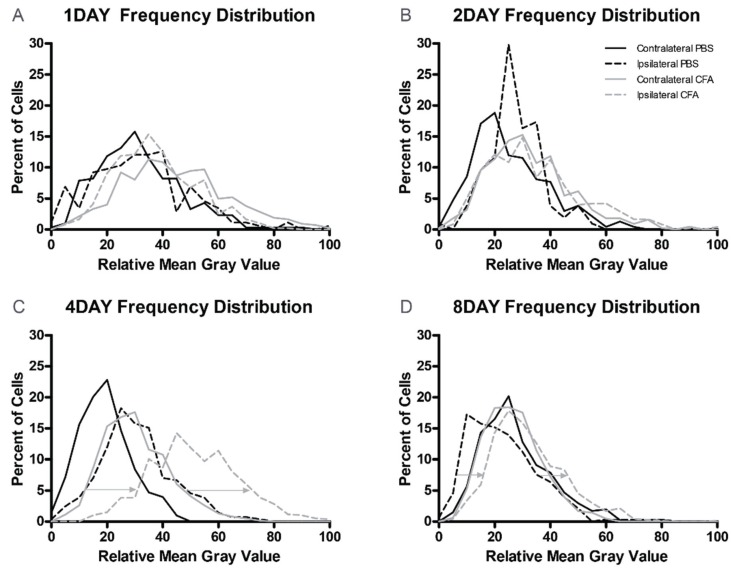
Frequency distributions for GLS-ir in L4 DRG neurons. (**A**,**B**) There were no obvious differences among the GLS-ir mean gray intensity (MGI) frequency distributions for ipsilateral or contralateral L4 DRG in PBS- or CFA-injected rats; (**C**) At day 4 AIA, there was a rightward shift (arrows) in the GLS-ir frequency distribution of the L4 DRG neuronal population ipsilateral to the CFA injection compared to the ipsilateral PBS injection; (**D**) At day 8 AIA, a slight right shift (arrows) remained in the GLS-ir frequency distribution of the L4 DRG neuronal population ipsilateral to the CFA injection compared to the ipsilateral PBS injection.

**Figure 4:**
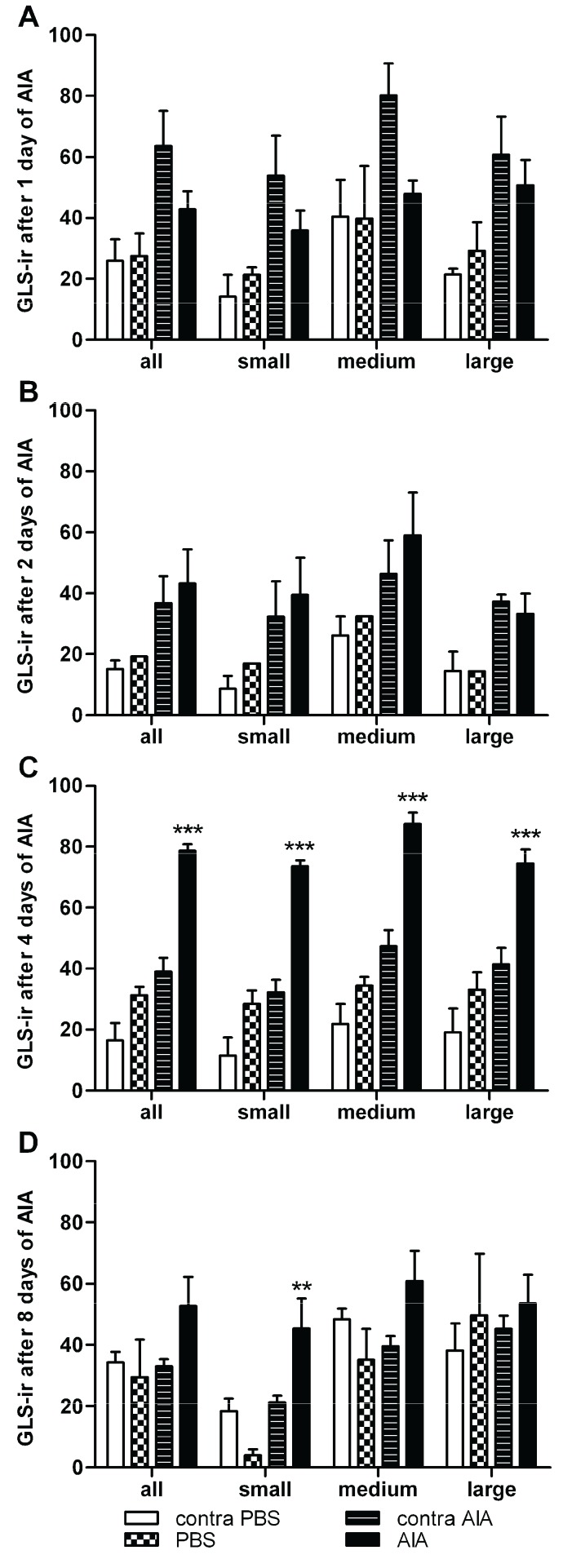
Image analysis of GLS-ir for small, medium, large, and all L4 DRG neurons. (**A**,**B**) No significant difference in GLS-ir occurred after one or two days of inflammation; (**C**) Elevation in GLS-ir occurred in all DRG neuronal sizes after four days of inflammation (*** *p* < 0.001; 121.4% overall increase); (**D**) GLS-ir elevation also occurred in small neurons after eight days of inflammation (** *p* < 0.01).

As in previous reports, all DRG neurons were immunoreactive for GLS (Figure 2) [14,15,18,20,25]. Representative images from the DRG ipsilateral to the PBS and CFA injections after one, two, four, and eight days of inflammation (Figure 2) showed a qualitative increase in GLS-ir in DRG neurons of AIA rats, especially after four days of AIA (Figure 2F). Frequency distributions of GLS-ir were similar among ipsilateral and contralateral PBS- and CFA-injected groups after one and two days of AIA (Figure 3A,B). At four and eight days AIA, there was an observable “rightward shift” in the frequency distribution of GLS-ir in the DRG neuron population ipsilateral to the CFA injection (Figure 3C,D). With quantitative image analysis, there was no significant difference in GLS-ir after one or two days of inflammation (Figure 4A,B). A robust elevation in GLS-ir, however, was evident in all sizes of DRG neurons after four days of inflammation (*p* < 0.001; Figure 4C; 121.4% overall increase) that persisted in small neurons after eight days of inflammation (*p* < 0.01; Figure 4D). After four days of inflammation, GLS-ir in small, medium, and large DRG neurons ipsilateral to CFA injection was approximately double that of DRG neurons ipsilateral to PBS injection.

### 3.4. GLS Western Blots

**Figure 5:**
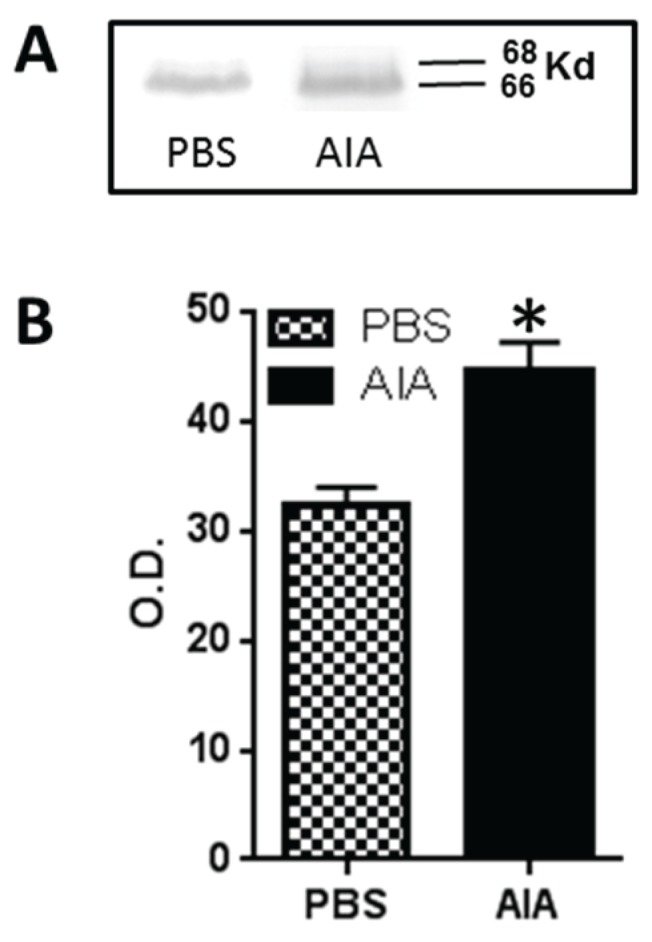
Western blot analysis of GLS from PBS- and CFA-injected rats at four days AIA. (**A**) Western blot of GLS from L4 DRG showed two bands, Mr 66,000 and Mr 68,000, corresponding to the two isoforms of GLS. There was an increase in GLS-ir in the 4 day AIA rats compared to PBS controls; (**B**) There was significant increase (38.1%) in the DRG from four day AIA rats compared to PBS-injected rats (* *p* < 0.05) (optical density (O.D.)—control: 32.34 ± 1.62; AIA: 44.67 ± 2.50).

Western blots of GLS from DRG demonstrated the presence of 66,000 and 68,000 molecular weight (M_r_) bands (Figure 5A) as previously reported for rat kidney and brain [30]. After four days of AIA, Western blots of ipsilateral DRGs demonstrated that the GLS-ir was significantly elevated (*p* < 0.05) when compared to control (optical density, O.D., control = 32.34 ± 1.62, AIA = 44.67 ± 2.50) representing a 38.1% increase (Figure 5B).

## 4. Discussion

Peripheral sensitization of DRG neurons during long-lasting inflammation initiates a sensitizing cascade along the pain pathway resulting in a chronic pain state. Both post-translational and expression dependent mechanisms are involved in peripheral sensitization; the former for initiation of sensitization during the acute stage of inflammatory pain and the latter for maintaining the sensitization during the chronic stage [11]. Many proteins involved in peripheral sensitization undergo both types of changes. While GLS does not have a phosphorylation site, its activity can be modulated by allosteric factors such as calcium and inorganic phosphate [32] and these concentrations increase in an activated peripheral or central axon terminal [7]. For example, increased terminal activity increases hydrolysis of ATP into ADP and inorganic phosphate [32] and increases calcium influx through voltage-gated calcium channels [33] and members of the TRP family [34]. Therefore, increased terminal activity during the acute stage of inflammation could increase GLS enzyme activity and account for the elevated amounts of glutamate reported for the skin [9,10], peripheral axons [35], and spinal cord dorsal horn within hours after the induction of inflammation [3,4].

In the current study, we have shown an elevation in GLS-ir indicating an increase in the content of GLS in DRG neurons after peripheral inflammation. There was a disparity in the amount of elevation with Western blotting (38%) *vs.* immunohistochemical image analysis (121%). This may be due to differences in antibody binding to GLS between the two techniques (denatured *vs.* lightly fixed protein). Additionally, the inclusion of satellite and Schwann cell protein to the overall protein for Western blotting may have diminished the percent increase in GLS compared to evaluating GLS-ir exclusively in the neuronal cytoplasm with image analysis [36]. Both techniques did show increases in GLS-ir and we interpret this as increased expression of GLS by DRG neurons, either by transcription dependent changes or enhanced translation of GLS protein. Based on the timescale of increased GLS production, it is possible that a retrogradely transported neurotrophic factor such as nerve growth factor (NGF) is responsible for this regulation. Retrograde NGF signaling also could account for subsequent anterograde transport out of newly synthesized GLS out of the cell body via mitochondrial axonal transport [37,38]. Moreover, NGF regulates expression of several other proteins important for nociception [25,39], and has the ability to affect GLS expression in DRG neurons [40,41,42] and retina [43].

Glutamate is implicated not only as a neurotransmitter released by nociceptive peripheral axon terminals, but also as a sensitizer of these terminals [7]. Injecting glutamate or glutamate receptor agonists sensitize nociceptors in *ex vivo* [44] studies and causes hyperalgesia *in vivo* in rodents [45,46,47,48,49,50,51,52,53,54] and humans [55]. The role of glutamate in mediating hyperalgesia during inflammation is evidenced by high glutamate levels in inflamed tissues [56,57] and the attenuation of hyperalgesia with glutamate receptor antagonists in inflammatory animal models and burn injury in humans [45,52,53,58,59]. Glutamate may exacerbate the neurogenic component of inflammation by further activation of neuropeptide release [60,61]. The numbers of glutamate receptors on peripheral axons also increases during inflammation [49,58], which could lead to an escalating cycle wherein the peripheral terminals are able to maintain their own sensitization and exacerbate chronic pain ([62]. Similarly, glutamate is involved in sensitization of dorsal horn neurons to afferent stimulation [63,64,65,66]. The presence of glutamate receptors on central axon terminals [67] could enhance central glutamate release via a positive feedback mechanism. Therefore, increased glutamate production may be involved in both peripheral and central sensitization mechanisms that culminate in chronic pain. The increase of GLS specifically in small diameter DRG neurons at 8 days AIA would correspond with a chronic pain mechanism originating from nociceptive DRG neurons. Future studies are directed toward evaluating GLS alterations during inflammation in DRG neurons with specific nociceptive markers, e.g., CGRP.

Since GLS is only one of many proteins involved in the glutamate-glutamine cycle, other proteins within the cycle may increase their activities and/or expressions in order to effectively increase the flux of glutamate through the cycle. Very little is known about the glutamate-glutamine cycle during inflammation, although many of these proteins have been localized to the DRG and peripheral nerve [20]. The neuronal glutamate transporter responsible for reuptake of glutamate, excitatory amino acid transporter 3 (EAAT3), localizes to primarily small diameter DRG neurons [21] and the glial glutamate transporter, EAAT1, localizes to satellite glial cells of the DRG [17]. Glutamine synthetase, the glial enzyme that converts glutamate to glutamine, not only serves as a specific marker for satellite glial cells in the rat DRG [20] and mouse trigeminal ganglia (TG) [68,69], but increases along with glutamine in satellite glial cells after peripheral inflammation [70]. Increases in GS and glutamine concur with the notion that multiple glutamate-glutamine cycle proteins and substrates increase in response to inflammation. Little is known concerning glutamine transporters in the DRG, but we have detected sodium-coupled neutral amino acid transporters (SNAT) 1 [71] and 2 [72] in DRG neurons. Vesicular glutamate transporters (VGLUTs) are necessary at sites of glutamate neurotransmission and VGLUT1 and 2 localize to different size classes of DRG and TG neuron cell bodies [73,74,75] in addition to the peripheral and central terminals [73,75,76,77]. Regulation of VGLUT1 and 2 levels at the cell body occurs after peripheral nerve crush and/or axotomy [73,78,79] and VGLUT2 expression appears to regulate glutamate release during neuropathic pain [80]. Information, however, about alterations of VGLUT during chronic inflammation is lacking. We hypothesize that post-translational and/or expression dependent mechanisms may act on some or all of the aforementioned proteins to increase glutamate production during inflammation and contribute to peripheral and central sensitization. Support for this hypothesis comes from the observations that pharmacological intervention of GLS with an irreversible inhibitor at the peripheral terminal [81,82] or glutamine transporter and GS inhibition in dorsal horn [83,84] provide analgesia during inflammation. Peripheral inhibition of GLS also decreases Fos activation in dorsal horn neurons after inflammation [60].

## 5. Conclusions

The present results indicate that an increase in GLS production occurs during the chronic stage of inflammatory pain. Further study of glutamate-glutamine cycle proteins both peripherally and centrally during inflammation is necessary to fully understand the role glutamate metabolism plays in peripheral and central sensitization. It is expected that such knowledge of glutamate metabolism will provide useful targets for chronic pain.
